# Supplementary material for: Association between Accelerated Biological Aging, Diet, and Gut Microbiome
Source: Microorganisms. 2024 Aug 20;12(8):1719. doi: 10.3390/microorganisms12081719 (PMC11357197; doi:10.3390/microorganisms12081719)
Supplement: Supplementary file 1 [file microorganisms-12-01719-s001.zip › microorganisms-3132921-supplementary.pdf]

Supplemental Table S1: Primers used to amplify the V4 region: .

v4.SA501	AATGATACGGCGACCACCGAGATCTACACATCGTACGTATGGTAATTGTGTGCCAGCMGCCGCGGTAA
v4.SA502	AATGATACGGCGACCACCGAGATCTACACACTATCTGTATGGTAATTGTGTGCCAGCMGCCGCGGTAA
v4.SA503	AATGATACGGCGACCACCGAGATCTACACTAGCGAGTTATGGTAATTGTGTGCCAGCMGCCGCGGTAA
v4.SA504	AATGATACGGCGACCACCGAGATCTACACCTGCGTGTTATGGTAATTGTGTGCCAGCMGCCGCGGTAA
v4.SA505	AATGATACGGCGACCACCGAGATCTACACTCATCGAGTATGGTAATTGTGTGCCAGCMGCCGCGGTAA
v4.SA506	AATGATACGGCGACCACCGAGATCTACACCGTGAGTGTATGGTAATTGTGTGCCAGCMGCCGCGGTAA
v4.SA507	AATGATACGGCGACCACCGAGATCTACACGGATATCTTATGGTAATTGTGTGCCAGCMGCCGCGGTAA
v4.SA508	AATGATACGGCGACCACCGAGATCTACACGACACCGTTATGGTAATTGTGTGCCAGCMGCCGCGGTAA
v4.SB501	AATGATACGGCGACCACCGAGATCTACACCTACTATATATGGTAATTGTGTGCCAGCMGCCGCGGTAA
v4.SB502	AATGATACGGCGACCACCGAGATCTACACCGTTACTATATGGTAATTGTGTGCCAGCMGCCGCGGTAA
v4.SB503	AATGATACGGCGACCACCGAGATCTACACAGAGTCACTATGGTAATTGTGTGCCAGCMGCCGCGGTAA
v4.SB504	AATGATACGGCGACCACCGAGATCTACACTACGAGACTATGGTAATTGTGTGCCAGCMGCCGCGGTAA
v4.SB505	AATGATACGGCGACCACCGAGATCTACACACGTCTCGTATGGTAATTGTGTGCCAGCMGCCGCGGTAA
v4.SB506	AATGATACGGCGACCACCGAGATCTACACTCGACGAGTATGGTAATTGTGTGCCAGCMGCCGCGGTAA
v4.SB507	AATGATACGGCGACCACCGAGATCTACACGATCGTGTTATGGTAATTGTGTGCCAGCMGCCGCGGTAA
v4.SB508	AATGATACGGCGACCACCGAGATCTACACGTCAGATATATGGTAATTGTGTGCCAGCMGCCGCGGTAA
v4.SA701	CAAGCAGAAGACGGCATACGAGATAACTCTCGAGTCAGTCAGCCGGACTACHVGGGTWTCTAAT
v4.SA702	CAAGCAGAAGACGGCATACGAGATACTATGTCAGTCAGTCAGCCGGACTACHVGGGTWTCTAAT
v4.SA703	CAAGCAGAAGACGGCATACGAGATAGTAGCGTAGTCAGTCAGCCGGACTACHVGGGTWTCTAAT
v4.SA704	CAAGCAGAAGACGGCATACGAGATCAGTGAGTAGTCAGTCAGCCGGACTACHVGGGTWTCTAAT
v4.SA705	CAAGCAGAAGACGGCATACGAGATCGTACTCAAGTCAGTCAGCCGGACTACHVGGGTWTCTAAT
v4.SA706	CAAGCAGAAGACGGCATACGAGATCTACGCAGAGTCAGTCAGCCGGACTACHVGGGTWTCTAAT
v4.SA707	CAAGCAGAAGACGGCATACGAGATGGAGACTAAGTCAGTCAGCCGGACTACHVGGGTWTCTAAT
v4.SA708	CAAGCAGAAGACGGCATACGAGATGTCGCTCGAGTCAGTCAGCCGGACTACHVGGGTWTCTAAT
v4.SA709	CAAGCAGAAGACGGCATACGAGATGTCGTAGTAGTCAGTCAGCCGGACTACHVGGGTWTCTAAT
v4.SA710	CAAGCAGAAGACGGCATACGAGATTAGCAGACAGTCAGTCAGCCGGACTACHVGGGTWTCTAAT
v4.SA711	CAAGCAGAAGACGGCATACGAGATTCATAGACAGTCAGTCAGCCGGACTACHVGGGTWTCTAAT
v4.SA712	CAAGCAGAAGACGGCATACGAGATTCGCTATAAGTCAGTCAGCCGGACTACHVGGGTWTCTAAT
v4.SB701	CAAGCAGAAGACGGCATACGAGATAAGTCGAGAGTCAGTCAGCCGGACTACHVGGGTWTCTAAT
v4.SB702	CAAGCAGAAGACGGCATACGAGATATACTTCGAGTCAGTCAGCCGGACTACHVGGGTWTCTAAT

v4.SB703 CAAGCAGAAGACGGCATACGAGATAGCTGCTAAGTCAGTCAGCCGGACTACHVGGGTWTCTAAT

v4.SB704 CAAGCAGAAGACGGCATACGAGATCATAGAGAAGTCAGTCAGCCGGACTACHVGGGTWTCTAAT

v4.SB705 CAAGCAGAAGACGGCATACGAGATCGTAGATCAGTCAGTCAGCCGGACTACHVGGGTWTCTAAT

v4.SB706 CAAGCAGAAGACGGCATACGAGATCTCGTTACAGTCAGTCAGCCGGACTACHVGGGTWTCTAAT

v4.SB707 CAAGCAGAAGACGGCATACGAGATGCGCACGTAGTCAGTCAGCCGGACTACHVGGGTWTCTAAT

v4.SB708 CAAGCAGAAGACGGCATACGAGATGGTACTATAGTCAGTCAGCCGGACTACHVGGGTWTCTAAT

v4.SB709 CAAGCAGAAGACGGCATACGAGATGTATACGCAGTCAGTCAGCCGGACTACHVGGGTWTCTAAT

v4.SB710 CAAGCAGAAGACGGCATACGAGATTACGAGCAAGTCAGTCAGCCGGACTACHVGGGTWTCTAAT

v4.SB711 CAAGCAGAAGACGGCATACGAGATTCAGCGTTAGTCAGTCAGCCGGACTACHVGGGTWTCTAAT

v4.SB712 CAAGCAGAAGACGGCATACGAGATTCGCTACGAGTCAGTCAGCCGGACTACHVGGGTWTCTAAT

Read 1 primer for V4 region

TATGGTAATTGTGTGCCAGCMGCCGCGGTAA

Read 2 primer for V4 region

AGTCAGTCAGCCGGACTACHVGGGTWTCTAAT

Index primer for V4 region

ATTAGAWACCCBDGTAGTCCGGCTGACTGACT
